# Disturbance-based management of ecosystem services and disservices in partial nitritation-anammox biofilms

**DOI:** 10.1038/s41522-022-00308-w

**Published:** 2022-06-08

**Authors:** Carolina Suarez, Christopher J. Sedlacek, David J. I. Gustavsson, Alexander Eiler, Oskar Modin, Malte Hermansson, Frank Persson

**Affiliations:** 1 Department of Chemistry and Molecular Biology, University of Gothenburg, Gothenburg, Sweden; 2 Division of Water Resources Engineering, Faculty of Engineering LTH, Lund University, Lund, Sweden; 3 University of Vienna, Division of Microbial Ecology, Centre for Microbiology and Environmental Systems Science, Vienna, Austria; 4 University of Vienna, The Comammox Research Platform, Vienna, Austria; 5 VA SYD, P.O. Box 191, SE-20121 Malmö, Sweden; 6 Sweden Water Research, Ideon Science Park, Scheelevägen 15, SE-22370 Lund, Sweden; 7 Section for Aquatic Biology and Toxicology, Centre for Biogeochemistry in the Anthropocene, Department of Biosciences, University of Oslo, Oslo, Norway; 8 Division of Water Environment Technology, Department of Architecture and Civil Engineering, Chalmers University of Technology, Gothenburg, Sweden

**Keywords:** Water microbiology, Microbial ecology

## Abstract

The resistance and resilience provided by functional redundancy, a common feature of microbial communities, is not always advantageous. An example is nitrite oxidation in partial nitritation-anammox (PNA) reactors designed for nitrogen removal in wastewater treatment, where suppression of nitrite oxidizers like *Nitrospira* is sought. In these ecosystems, biofilms provide microhabitats with oxygen gradients, allowing the coexistence of aerobic and anaerobic bacteria. We designed a disturbance experiment where PNA biofilms, treating water from a high-rate activated sludge process, were constantly or intermittently exposed to anaerobic sidestream wastewater, which has been proposed to inhibit nitrite oxidizers. With increasing sidestream exposure we observed decreased abundance, alpha-diversity, functional versatility, and hence functional redundancy, among *Nitrospira* in the PNA biofilms, while the opposite patterns were observed for anammox bacteria within *Brocadia*. At the same time, species turnover was observed for aerobic ammonia-oxidizing *Nitrosomonas* populations. The different exposure regimens were associated with metagenomic assembled genomes of *Nitrosomonas*, *Nitrospira*, and *Brocadia*, encoding genes related to N-cycling, substrate usage, and osmotic stress response, possibly explaining the three different patterns by niche differentiation. These findings imply that disturbances can be used to manage the functional redundancy of biofilm microbiomes in a desirable direction, which should be considered when designing operational strategies for wastewater treatment.

## Introduction

Disturbances are common phenomena affecting both natural and engineered systems leading to changes in biodiversity, as well as impacting ecosystem functioning^1^. Protection against the loss of ecosystem services even when ecosystems are exposed to disturbances can be provided by highly diverse communities, as stated by the insurance hypothesis^2^. This is because functional redundancy, a direct result of biodiversity, is predicted to provide resilience and resistance against environmental change^3,4^. Furthermore, biodiversity is linked to multifunctionality and the capability of a community or population to provide a multitude of ecosystem services^5–7^. In highly diverse microbial systems such as soils, sediments and wastewater treatment bioreactors, a high degree of multifunctionality and functional redundancy should consequently lead to high resilience and a resistant supply of ecosystem services. These are preferred features for many ecosystems, protecting them from anthropogenically induced changes^8,9^. However, in engineered systems certain ecosystem services are unwanted (so called disservices)^10,11^. Functional redundancy of disservices within an engineered system can hinder the tuning of these systems towards the desired reactor function.

In wastewater treatment plants (WWTPs), enhanced biological nitrogen removal is used to reduce the amount of reactive nitrogen in the effluent, which otherwise would lead to impaired water quality and eutrophication in recipient waterways^12^. The recent focus on energy neutrality at WWTPs has promoted processes based on nitritation. This can be achieved with partial nitritation-anammox (PNA) by maintaining two ecosystem services; aerobic oxidation of half of the influent ammonia to nitrite by ammonia-oxidizing bacteria (AOB), and anaerobic oxidation of the remaining ammonia with reduction of nitrite to form nitrogen gas by anaerobic ammonia-oxidizing (anammox) bacteria^13^. In PNA, further oxidation of nitrite to nitrate by nitrite-oxidizing bacteria (NOB) is undesired^14^ and hence an ecosystem disservice. The two steps of aerobic and anaerobic ammonium oxidation can take place either in separate reactors (two-stage) or in the same reactor (one-stage). Because of the slow growth rate of the AOB and anammox bacteria, biofilm systems are commonly used to maintain the biomass in the WWTP bioreactors^15^. Moreover, biofilms provide oxygen gradients allowing the coexistence of the AOB and anammox bacteria in the same biofilm, in the case of one-stage reactors^16^.

AOB and NOB belong to multiple genera, where *Nitrosomonas*, *Nitrospira* and *Nitrotoga* are the most common in WWTPs. Furthermore, multiple subpopulations are often reported to coexist for the AOB *Nitrosomonas*^17,18^ and the NOB *Nitrospira*^19,20^. This microdiversity within populations might represent ecotypes, closely related populations that can occupy different ecological niches^21^. For example, closely related *Nitrosomonas* and *Nitrospira* populations are known to differ in their preferences for substrate concentration^22–24^ and temperature^23,25^, as well as their ability to use alternative energy sources such as urea, cyanate, formate, and hydrogen^24,26–28^. In addition, some *Nitrospira* are nitrite oxidizers, while other closely related *Nitrospira* are capable of complete ammonia oxidation to nitrate^29–32^. In the case of anammox bacteria, microdiversity within wastewater treatment microbiomes is less documented but has been demonstrated in marine ecosystems^33,34^. It is also evident that ecotypes within *Brocadia*, common in anammox wastewater treatment reactors, differ in their substrate preferences and responses to reactor operation^35,36^. As opposed to the autotrophic processes of aerobic ammonia- and nitrite oxidation, and anammox, denitrification is a process carried out by a large number of taxa, distributed across multiple phyla, with considerable metabolic versatility^37^.

PNA has been implemented at many WWTPs to treat the dewatering liquor from anaerobic digestion of sludge (“sidestream water”), which is warm (>25 °C) and ammonium rich (>500 mg NH_4_-N/L)^13^. However, treating municipal wastewater (“mainstream water”) that has less ammonium (<100 mg NH_4_-N/L) has been challenging, in particular for temperate regions with cold water (<15 °C)^15,38^. Here, both modeling approaches and experimental results have shown that it is difficult to simultaneously maintain high activities of AOB and anammox bacteria and inhibit unwanted NOB activity^15,39^.

Multiple strategies have been used to inhibit NOB in the mainstream, such as short solids retention time, dissolved oxygen limitation, intermittent aeration, free ammonia exposure, free nitrous acid exposure and acidic nitritation^38,40–42^. Recent studies have shown that it is important to understand how NOB populations respond to changes in environmental conditions to devise effective inhibition strategies^20,43^. Exposing mainstream communities to sidestream wastewater has previously been proposed as a method to inhibit NOB^40,44^. The inhibition could occur due to multiple mechanisms, such as increased competition for nitrite and oxygen with anammox bacteria and AOB, respectively, and the presence of higher free ammonia concentrations^45–47^. Therefore, a disturbance management with sidestream exposure may reduce the biodiversity and functional redundancy of NOB in PNA bioreactors. However, for disturbance management with sidestream exposure to be a viable approach, the functional diversity of AOB and anammox bacteria must not be negatively affected. Shifts in the composition of NOB, AOB, and anammox bacteria can be expected, but the effects on abundance, biodiversity, and functional redundancy are unknown. It is also important to consider the frequency and intensity of disturbances, which in bioreactors have been shown to have impact in microbial community composition and ecosystem function^48–50^.

**Fig. 1:**
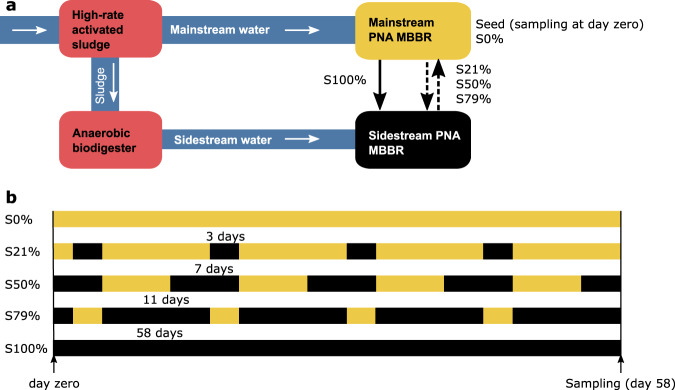
Scheme of the PNA MBBRs at the Sjölunda WWTP and the experimental design. **a** Layout of the WWTP and the PNA MBBRs. White arrows indicate the water flow. The black arrows indicate transfer of biofilm carriers between the PNA MBBRs (yellow/black). **b** Biofilm carriers were either kept in the mainstream PNA MBBR (S0%), moved to the sidestream PNA MBBR (S100%), or moved between the mainstream and the sidestream in 2-week periods according to the time schedule (S21%, S50% and S79%). Initial samples from the mainstream PNA MBBR, at time zero were also analyzed (Seed). The 2-week rotation periods were repeated four times over the course of 58 days.

At the Sjölunda WWTP (Malmö, Sweden), one-stage mainstream and sidestream pilot PNA reactors harbored biofilm communities with *Nitrospira*, *Nitrosomonas* and *Brocadia* populations, which have been previously characterized to contain various degrees of microdiversity^51^. In this study, we designed a disturbance experiment where mainstream PNA biofilms during winter were repeatedly exposed to sidestream wastewater for specific time periods and compared the treatment with PNA biofilms kept in the mainstream bioreactor (Fig. 1). We ask if such a disturbance regimen could be utilized to shift the microbial community of biofilms towards a desired state. To address this question, we describe the effects of a temporal gradient of sidestream exposure on the abundance, microdiversity, and functional diversity of NOB, AOB, anammox- and denitrifying bacteria.

Our main hypothesis is that the effect of sidestream wastewater would differ across functional groups. More specifically, we hypothesize that the microdiversity of *Nitrospira, Nitrosomona*s and *Brocadia* will shift due to the disturbances, but that the denitrifying microbiome, which is dispersed across multiple phyla, will be less affected by the disturbances.

## Results and discussion

Biofilm carriers from a mainstream PNA moving bed biofilm bioreactor (MBBR) were transferred to sidestream wastewater in another MBBR at time intervals corresponding to 21%, 50%, 79% and 100% in the sidestream (S21%, S50%, S79% and S100%) over the course of 58 days. A mainstream control (S0%) as well as the initial seed sample (Seed, time zero) were also included in the study (Fig. 1).

### Rare taxa were particularly sensitive to sidestream wastewater

In PNA biofilms, nitrifiers are restricted to a thin oxic layer at the surface of the biofilm, and hence their relative abundance in the biofilm is low^16,51^; therefore, beta-diversity metrics, sensitive to rare taxa, are well-suited to elucidate their distribution patterns. For metrics based on Hill-numbers, this sensitivity can be controlled with the q parameter (diversity order). When *q* = 0, which is a presence-absence metric, the index is insensitive to the relative abundances of taxa (equivalent to the Sørensen index) while for increasing *q*, the index is more sensitive to abundant taxa^52,53^.

**Fig. 2:**
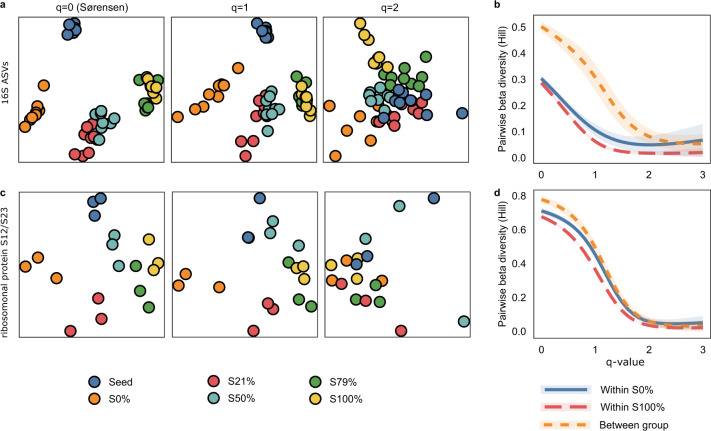
Beta-diversity of the biofilm communities. Beta-diversity of 16S rRNA ASVs (**a**, **b**) and of the metagenomics dataset using the ribosomal protein S12/S23 (**c**, **d**), based on Hill numbers. **a**, **c** PCoA when *q* = 0 (i.e. the Sørensen index), *q* = 1 or *q* = 2; each circle corresponds to a biofilm carrier. **b**, **d** Within group and between group beta-diversity for biofilms kept in the mainstream (S0%) and moved to the sidestream (S100%) at different *q*-values; the lines show the average pairwise beta-diversity and the shaded areas show 95% confidence intervals.

The entire PNA mainstream biofilm community appears to be sensitive to sidestream wastewater, as we observed differences between treatments for both rare and abundant taxa in the 16S dataset (PERMANOVA *q* = 0, 1 & 2; *p* < 0.001; F_4,40_ = 7.4, 42 & 41, respectively; Fig. 2a). In fact, there was a beta-diversity gradient between S0% and S100%, corresponding to the extent of sidestream exposure (Fig. 2a). Between group beta-diversity was the highest at *q* = 0 and was also higher than within group dissimilarity at low q-values (Fig. 2b), implying that rare taxa were more sensitive to sidestream exposure than abundant taxa. Similar beta-diversity patterns were seen in the metagenome dataset (Fig. 2c, d), with differences for rare taxa (PERMANOVA *q* = 0 and *q* = 1; *p* < 0.001; F_4,10_ = 1.3 & 1.6, respectively), but not the abundant ones (PERMANOVA *q* = 2; *p* = 0.16; F_4,10_ = 1.5).

### Response in alpha-diversity and abundance to sidestream exposure varies between taxa

**Fig. 3:**
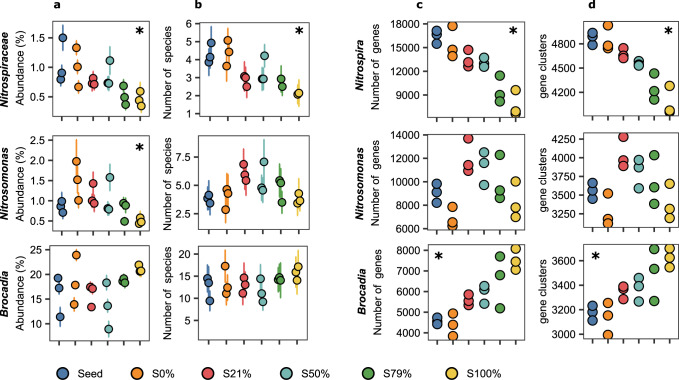
Abundance and richness of *Nitrospira* (top), *Nitrosomonas* (middle), and *Brocadia* (bottom) in the metagenome dataset. **a** Relative abundances. **b** The number of species. **c** The number of genes. **d** Number of pangenome gene clusters. **a**, **b** singleM data; circles show average values of 14 marker genes, error bars indicate 95% confidence intervals. **c**, **d** Kaiju data. Note that Kaiju uses NCBI taxonomy, while singleM uses GTDB taxonomy; in the latter the genus *Nitrospira* is classified as the family *Nitrospiraceae*. Pearson correlation was estimated for sidestream exposure from 0 to 100%, for the singleM average (**a**, **b**) and the Kaiju values (**c**, **d**); an asterisk (*) is used to show significant correlations (*p* < 0.05).

The relative abundance and alpha-diversity of *Nitrospiraceae* in the biofilms decreased with sidestream exposure, as assessed by marker genes (Fig. 3a, b). As an alternative to the marker gene-based assessment of species diversity, we assigned taxonomy to individual genes in the metagenome assembly, and thus estimate a rough distribution of those genes across samples. From this analysis, it was clear that the number of *Nitrospira* genes also decreased with increasing sidestream exposure (Fig. 3c).

In contrast to the *Nitrospira*, for the AOB *Nitrosomonas*, a higher relative abundance, alpha-diversity and number of genes occurred at intermittent exposure to sidestream water (S21–S79%; Fig. 3), akin to predictions by the intermediate disturbance hypothesis^48,54^. Among the anammox bacteria (*Brocadia*) an average of ~15 genotypes were detected across all treatments, but the number of *Brocadia* genes steadily increased with increasing sidestream exposure (Fig. 3). Thus, negative (*Nitrospira/Nitrospiraceae*), intermediate (*Nitrosomonas*) and positive (*Brocadia*) effects on intra-genera diversity were clearly observed in response to the varying levels of disturbance.

The pangenome, coined to refer to all the genes that can be found in a clade including the core and all accessory genes^55,56^, is assumed to be responsible for niche differentiation and allows the discrimination of different ecotypes, defined as populations of cells adapted to a given ecological niche. We grouped all genes taxonomically classified as *Nitrospira* into gene clusters, which, accordingly, could be considered as the *Nitrospira* pangenome (Fig. 3d). We show that the number of *Nitrospira* gene clusters has a downward trend, suggesting a decrease in functional potential with increased sidestream exposure. This reduction in functional redundancy within *Nitrospira* is beneficial when managing a disservice, as is the case of nitrite oxidation in PNA processes. Similar observations were made for *Nitrospira* in a recent study in nitrifying activated sludge^57^. The opposite pattern occurred for *Brocadia*, leading to increased diversity (Fig. 3d), and thus enhanced redundancy in the functional group of anammox bacteria. For *Nitrosomonas*, the number of gene clusters followed the pattern of the alpha-diversity, i.e., higher numbers at intermediate disturbances.

At the Sjölunda WWTP, relative nitrate production was higher in the mainstream PNA MBBR than in the sidestream PNA MBBR^51^. In addition, other studies have shown that for mainstream communities, exposure to sidestream water leads to nitrite accumulation due to the inhibition of NOB^20,40,43,44^. Together, with the results from this report, this data highlights how sidestream wastewater disturbance management regimens can be used to inhibit NOB populations in PNA biofilms and preserve AOB and anammox.

### Distinct *Nitrosomonas* populations were present in the sidestream and mainstream

**Fig. 4:**
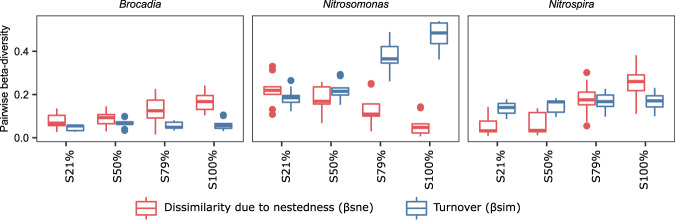
Pairwise beta-diversity between S0% and other treatments using the Kaiju data from Fig. 3C. The presence-absence Sørensen index (*q* = 0) was estimated as its two components: species turnover (i.e. the Simpson index) and dissimilarity due to nestedness. In the plot, center line: median, box limits: upper and lower quartiles, whiskers: 1.5 inter-quartile range, dots: outliers.

Differences between communities (beta-diversity) can arise because of species turnover (when one species is replaced by another) or nestedness (when one community is a subset of the other)^58^. The turnover/nestedness framework has been used extensively in community ecology^59^ and has contributed to our understanding of biofilm assembly^60^. For *Nitrospira* and *Brocadia* genes, beta-diversity between S0% and S100% was increasingly caused by dissimilarity due to nestedness (Fig. 4). For *Nitrospira*, alpha-diversity decreased with sidestream exposure (Fig. 3) making the *Nitrospira* sidestream (S100%) community a subset of the mainstream (S0%) community. Opposite patterns were observed for *Brocadia* where the mainstream community was a subset of the sidestream one. Notably, this was not the case for *Nitrosomonas*, where the beta-diversity between S0% and S100% was increasingly caused by turnover (Fig. 4). Thus, different *Nitrosomonas* populations were present in S0% and S100%. Similar results were observed in the 16S dataset, where a single *Nitrosomonas* ASV was dominant in the mainstream wastewater and its abundance was lower in all samples exposed to sidestream wastewater, while another *Nitrosomonas* ASV dominated samples exposed to sidestream wastewater (Supplementary Fig. 1).

### Distinct *Nitrosomonas* MAGs were observed in the sidestream and mainstream

**Fig. 5:**
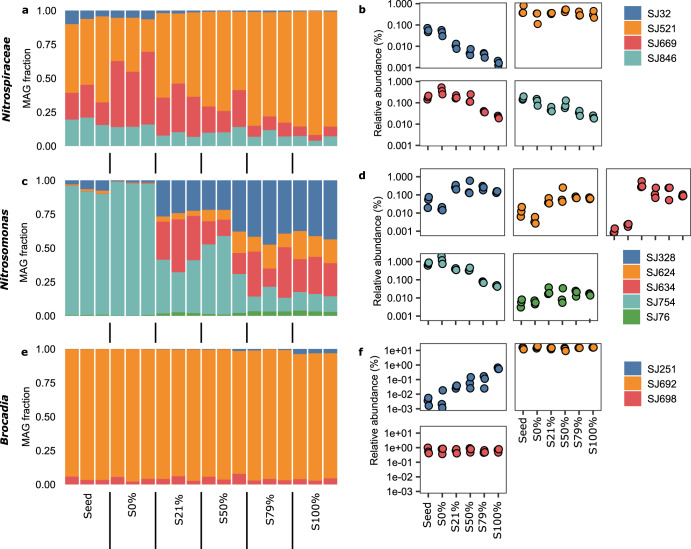
Abundance of MAGs classified as *Nitrospiraceae*, *Nitrosomonas* and *Brocadia*. **a**, **c**, and **e** Relative abundance within the group. **b**, **d**, and **f** Relative abundance against all the bins in the metagenome.

To characterize the populations that were responsible for beta-diversity, metagenomic reads were assembled and resulting contigs were binned, resulting in 430 metagenome assembled genomes (MAGs). Of these, five MAGs were affiliated to *Nitrosomonas* (Fig. 5). For four of them, their completeness ranged from 88 to 97%. A fifth MAG, SJ328, was only 58% complete (File S1). The MAG SJ754 made up more than 90% of the *Nitrosomonas* community in S0% but decreased sharply in abundance with increasing sidestream exposure to about ~13% in the S100% samples (Fig. 5C, D). With increasing sidestream exposure the relative abundance of the other four *Nitrosomonas* MAGs increased.

Genome reconstruction suggests that the AOB differed in their tolerance to osmotic stress, ability to use urea or hydrogen, tolerance to oxygen limiting conditions, and ability to fix carbon during low and fluctuating CO_2_ concentrations (See supporting information for details). These differences could lead to niche differentiation between the AOB and possibly explain the observed AOB turnover.

Alternatively, or concurrently, fluctuations in abundance of the AOB MAGs could be due to stochastic processes rather than niche differentiation^18^, as migration of AOB from upstream to downstream processes is known to occur in WWTPs^61^. Thus, the high abundance of SJ754 in the mainstream samples could have been caused by continuous immigration from the upstream activated sludge, akin to the mass-effect perspective in the metacommunity framework^62^. However, given the low sludge retention time (2–3 days) in the preceding high-rate activated sludge with no signs of nitrification in the investigated period, such stochastic effects were likely negligible for both the *Nitrosomonas* and the *Nitrospira* communities.

### A lineage I *Nitrospira* was resistant to sidestream exposure

In general, the *Nitrospira* populations were sensitive to sidestream exposure, with the relative abundance of three of the four detected MAGs decreasing in abundance with increasing sidestream exposure (Fig. 5b). These results align with the observed decrease in *Nitrospira* alpha-diversity (Fig. 3). The sole exception was SJ521, a *Nitrospira* lineage I NOB, whose relative abundance remained the same regardless of sidestream exposure (Fig. 5b).

As SJ521 was the dominant *Nitrospira* MAG in S100%, it was hypothesized that this MAG would encode genes providing this species with niche differentiating traits absent in the other *Nitrospira* and thus provide a competitive advantage. Previously, several *Nitrospira* species have been shown to possess genes that enable use of alternative energy sources such as ammonium, formate, or hydrogen and are able to engage in reciprocal feeding with AOB through the turnover of urea or cyanate^26–28^.

Three of the four *Nitrospira* MAGs, including SJ521, encode a very similar suite of genes for these alternative metabolisms (File S1). However, the dominant MAG SJ521, is the only one that contains a Na^+^/H^+^ antiporter complex, which can regulate cytoplasmic pH and/or Na^+^ concentration and confer halotolerance^63^. To date, the only *Nitrospira* genomes observed to encode this kind of antiporter complex have been found in highly saline/alkaline environments^64^. Interestingly, similar Na^+^/H^+^ antiporter complexes were also found in four of the five *Nitrosomonas* MAGs (File S1).

The presence of halotolerant populations of *Nitrospira* and *Nitrosomonas* after sidestream exposure is unexpected, as the concentrations of Na^+^ ions were the same in the mainstream and sidestream (Supplementary Table S1). Nonetheless, the conductivity, which is a measure of the total concentration of ions in the water, was more than five-fold higher in the sidestream, indicating a higher osmotic stress in the sidestream. In this environment, adaptations to osmotic stress might offer a competitive advantage to halotolerant populations. Hence, it is possible that uncharacterized adaptations to osmotic stress in *Nitrospira* SJ521 could explain the persistence of this population.

### Abundance of *Brocadia fulgida* increased with sidestream exposure

Three *Brocadia* MAGs were present in the PNA biofilms (Fig. 5e). SJ692 was the dominant anammox MAG in the dataset, and its abundance did not change with sidestream exposure.

SJ251 had its highest abundance in the S100% samples (Fig. 5f), which explains the increase in functional diversity of anammox genes with sidestream exposure (Fig. 3c). Unlike the other MAGs, it has two pathways to incorporate acetate (See Supplementary Results and Discussion). Anaerobic biodigesters were located upstream of the sidestream reactor which resulted in much higher concentrations of volatile fatty acids (VFA) (196 mg COD/L) in the sidestream than the mainstream feed (< 20 mg COD/L). Due to the availability of acetate and other VFA, SJ251 might have a competitive advantage that differentiates their niche from the other anammox bacteria and allows them to prosper in the sidestream.

### Genes for denitrification were ubiquitous in the PNA biofilms

To test if sidestream exposure had an effect on the abundance of denitrifiers, we estimated the overall abundance of MAGs having the genes *nirK*, *nirS*, *norB* and *nosZ*. The effect varied among the denitrifying genes. Sidestream exposure and abundance did not correlate for MAGs with *nirS* and *norB*, while there was slight increase in abundance of MAGs with *nosZ* and *NirK* (Supplementary Fig. 2). In addition, shifts were observed within the distribution of MAGs having *nirK*, *nirS*, *norB* and *nosZ* (Supplementary Fig. 3–6). Since denitrification is a trait with a wide phylogenetic distribution, it is likely to be more resistant to disturbances than traits with a narrow phylogenetic distribution like nitrification and anammox

Potential links in abundance between some AOB MAGs (SJ328, 624, 634) and denitrifiers were found, and for *Nitrosomona*s SJ754 its abundance was linked to *Nitrospira* SJ669 and SJ846 (Supplementary Fig. 7). If they are co-dependent, inhibition of *Nitrosomonas* SJ754 alone, might explain the decrease in abundance of *Nitrospira* SJ669 and SJ846 or vice versa. This may have importance for successful management of PNA biofilms.

In summary, this study shows that it is possible to manipulate the microbial community of a mature biofilm towards a desired state. With increased sidestream wastewater exposure, the functional versatility of PNA biofilms, and hence their functional redundancy, decreased for the unwanted functional group (NOB), and increased for the wanted functional groups (AOB and anammox bacteria). The observed responses to the disturbances demonstrate that exposure of sidestream wastewater has potential as a tool to regulate PNA biofilm communities. Our metagenomic analyses show that the pangenomes of nitrifiers and anammox bacteria included genes for osmotic stress response, organic matter and inorganic nitrogen usage. This provides not only multifunctionality but also functional redundancy, and thus resistance to the loss of services and disservices in ecosystems. For WWTPs, our findings imply that knowledge about the versatility of pangenomes should be considered when designing long-term operational strategies. Although we observed a range of effects of sidestream exposure on the *Nitrosomonas*, *Nitrospira*, and *Brocadia* populations, the detailed mechanisms remain unclear. Future studies using other approaches, like metatranscriptomics and activity measurements, may shed further light on the stress response to sidestream exposure.

## Methods

### Study design

Two parallel pilot PNA MBBRs at the Sjölunda WWTP (Malmö, Sweden) were fed with either mainstream wastewater from a high-rate activated sludge plant for organic carbon removal or sidestream wastewater, i.e. sludge liquor from anaerobic sludge digestion. Their volumes were 2.6 m^3^ and 1.5 m^3^, respectively. Both bioreactors were filled with biofilm carriers (K1®, Veolia Water Technologies AB—AnoxKaldnes, Lund, Sweden), which offer a protected biofilm surface. The reactors are described in detail elsewhere^51^. Conditions in the bioreactors are shown in Table 1.

**Table 1:** Conditions in the MBBRs during the period of investigation.

	Mainstream	Sidestream
Reactor NH_4_^+^ (mg N L^−1^)	10 ± 3.0	73 ± 24
Reactor NH_3_ – FA (mg N L^−1^)	0.04 ± 0.02	0.6 ± 0.2
Reactor NO_2_^−^ (mg N L^−1^)	0.45 ± 0.09	4.6 ± 0.7
Reactor HNO_2_ – FNA (mg N L^−1^)	9 ± 2·10^−5^	7 ± 1·10^−4^
Reactor NO_3_^−^ (mg N L^−1^)	3.2 ± 1.1	140 ± 28
Temperature (°C)	15 ± 1.4	25 ± 2.2
Dissolved Oxygen (mg L^−1^)	2.0 ± 0.2	1.3 ± 0.7

In this study, biofilm carriers were transferred between the mainstream and sidestream MBBRs at varying time intervals over 58 days. To differentiate these carries from others in the MBBRs, the biofilm carriers sampled in this study were kept isolated from the other carriers in the MBBRs in cylindrical cages. A steel mesh bottom of the cages allowed water circulation (immersed volume 2.5 L, 30% filling). All the cages were filled with biofilm carriers from the mainstream MBBR on November 11, 2015 and sampled on January 7, 2016. In addition, samples were taken directly from the mainstream PNA MBBR at the start of the experiment on November 11, before filling the cages. These samples are referred as Seed.

The layout of the study is described in Fig. 1. One cage with biofilm carriers was kept in the mainstream MBBR the entire experimental period, and hence spent zero time in the sidestream MBBR (S0%). Another cage was put in the sidestream MBBR the entire period (S100%). Three other cages were moved between the mainstream- and sidestream MBBRs at varying time intervals over the 58 days. Cage S21% spent 21% of its time in the sidestream MBBR in cycles of 3 days in the sidestream MBBR and 11 days in the mainstream MBBR; cage S50% had cycles of 7 days in mainstream MBBR and 7 days in sidestream MBBR; and cage S79% had cycles of 3 days in mainstream MBBR and 11 days in sidestream MBBR.

### Biofilm sampling and DNA extraction

For each treatment (the five cages and the Seed samples), DNA was separately extracted from nine biofilm carriers. The carriers were snap-frozen in an ethanol-dry ice mixture immediately at sampling, kept frozen in dry ice during transportation and then stored at −80 °C. The biofilm was removed from the carrier compartments and added to the lysis matrix tube E (MP biomedicals, Santa Ana, CA, USA) with 800 µl of lysis solution of the ZR-duet MiniPrep kit (Zymo Research). Mechanical disruption of the biofilm was done with a FastPrep-24 5 G (MP biomedicals) at speed 6 for 40 s. Subsequent steps of the DNA extraction were done with the ZR-duet kit according to the manufacturer’s instructions.

### 16S amplicon sequencing

Amplicon sequencing of the 16S rRNA V4 region (16S) was conducted for each of the nine DNA extractions in each treatment, resulting in 54 samples being sequenced. PCR amplification was done with primers the 515ʹF^65^ and 806 R^66^, using dual indexing of the primers^67^. PCR products were purified with Ampure XP (Beckman Coulter, Brea, CA, USA), and PCR amplicons were then pooled in equimolar amounts. Sequencing was carried out on an Illumina MiSeq using the MiSeq Reagent Kit v3 (Illumina, San Diego, CA, USA). DADA2 version 1.16^68^ was used to infer amplicon sequence variants (ASVs), with settings *pool* = *TRUE*. SILVA 138^69^ was used for taxonomic classification of the 16S amplicons.

### Shotgun metagenomics

For each treatment, three samples were selected for shotgun metagenomics, resulting in a total of 18 sequenced samples. Libraries were prepared with a TruSeq PCR-free kit (Illumina). Samples were sequenced on a NovaSeq 6000 with a 2 × 151 setup, resulting in 800 million pair-reads. Low quality reads and adapters were removed with Trimmomatic v0.39^70^. Co-assembly of short reads was done with Megahit^71^ using the *--presets meta-large* setting, resulting in 1,975,712 contigs of ≥1 kb, and N50 of 4375 bp. Reads were mapped to the co-assembly using Bowtie2 v2.3.5^72^. Protein coding sequences in all the assembled contigs were identified with Prodigal v.2.6.3^73^, using the meta prediction mode.

### Diversity analysis

SingleM (https://github.com/wwood/singlem) was used to create an OTU-like table of 14 single copy marker genes, which was used to estimate alpha- and beta-diversity in the metagenomics dataset. After subsampling to even depth, alpha-diversity was estimated as species richness for both the ASVs, based on the 16S amplicon sequencing, and the metagenomics marker genes.

Using the R package hilldiv^74^, dissimilarity indices were calculated based on partitioning of Hill numbers^75^ with a Sørensen-type overlap for both the 16S dataset and the metagenomics one. Beta-diversity was estimated from *q* = 0 to *q* = 3 in 0.1 intervals. Among samples taken at day 58, permutational multivariate analysis of variance (PERMANOVA)^76^ was used to test for significant differences between group centroids.

Kaiju^77^ was used for taxonomic classification of assembled genes against the NCBI BLAST nr database. Richness (number of classified genes) was estimated for *Nitrosomonas*, *Nitrospira* and *Brocadia*. Using the Kaiju data, the presence-absence Sørensen index, and its two components, turnover and dissimilarity due to nestedness^58^, were also estimated. To identify gene clusters, the proteins classified as *Nitrosomonas*, *Nitrospira* and *Brocadia* were aligned against each other with DIAMOND^78^ using --ultra-sensitive parameter, followed by clustering with MCL^78^, with an inflation factor of 8.

Hidden Markov models (HMM) from the FunGene database^79^ were used to search for AOB, *Nitrospira* or Archaea *amoA* in the metagenomics assembly with HMMER 3.3 (hmmer.org). The lowest E-value and highest bit score always corresponded to the amoA_AOB profile. In addition, the recovered *amoA* sequences were analyzed with an LG + F + G4 phylogenetic tree.

### Metagenome assembled genomes

Metagenomic binning was done in MetaBAT2^80^, resulting in 877 bins. Completeness, contamination, and relative abundance of the bins was estimated with CheckM^81^. 430 metagenome assembled genomes (MAGs) had more than 50% completion and less than 10% contamination. Taxonomic classification of these MAGs was done using GTDB-Tk^82^ with the GTDB r89 taxonomy^83,84^. To study evolutionary relationships of the *Nitrosomonas*, *Nitrospira* and *Brocadia* MAGs, the MAGs were compared with genomes from the NCBI assembly database. Phylogenomic trees based on single-copy genes were made with GtoTree v1.2.1^85^, using the Betaproteoabacteria HMM-set (203 genes) for *Nitrosomonas*, and the Bacteria HMM-set (74 genes) for *Nitrospira* and *Brocadia*. The multiple sequence alignment from GtoTree was used to generate maximum likelihood phylogenetic trees in IQ-TREE v.2.0.3^86^ with 1000 rapid bootstrap replicates; a substitution model for each gene in the alignment was chosen with ModelFinder^87^ using the partition files generated by GtoTree. Average nucleotide identity (ANI) estimations were done with FastANI^88^. The online version of eggNOG-mapper^89,90^ was used to annotate the *Nitrosomonas*, *Nitrospira* and *Brocadia* genomes. The *Nitrosomonas* and *Nitrospira* genomes were compared to each other and against previously sequenced nitrifiers using the integrated microbial genomes (IMG) database and the comparative analysis system^91^, as well as Genoscope^92^. With HMMER we searched for the presence of the genes *amoA*, *nirS*, *nirK*, *norB* and *nosZ* in all the MAGs. Network analysis based on the relative abundance of the MAGs was carried out using the R package SPIEC-EASI^93^ with the glasso method (lambda.min.ratio = .01, nlambda = 30, rep.num = 100).

### Salinity measurements

Three water samples from sidestream and mainstream at the Sjölunda WWTP were collected in January 2021. Conductivity was measured using a CO11 conductivity sensor (VWR, Radnor, Pennsylvania, United States). The concentration of Na^+^, Cl^−^, NH_4_^+^, K^+^, PO_4_^3−^, Mg^2+^, Ca^2+^ and SO_4_^2−^ was measured with an ICS-900 ion chromatograph (Dionex Sunnyvale, California, USA).

### Reporting summary

Further information on research design is available in the Nature Research Reporting Summary linked to this article.

## Supplementary information

Supplementary material

File S1

Reporting Summary Checklist

## Data Availability

Amplicon sequencing reads, raw shotgun metagenomics reads, and metagenome assembled genomes (MAGs) are available at NCBI under bioproject PRJNA611787. All data generated or analyzed during this study will be available upon request to the corresponding author.

## Code availability

Computer code used in this study is available upon request from the first author.
