# Supplementary material for: Disturbance-based management of ecosystem services and disservices in partial nitritation-anammox biofilms
Source: NPJ Biofilms Microbiomes. 2022 Jun 8;8:47. doi: 10.1038/s41522-022-00308-w (PMC9178042; doi:10.1038/s41522-022-00308-w)
Supplement: Supplementary file 1 — Supplementary material [file 41522_2022_308_MOESM1_ESM.pdf]

SUPPLEMENTARY INFORMATION FOR:

DISTURBANCE-BASED MANAGEMENT OF ECOSYSTEM SERVICES AND DISSERVICES IN PARTIAL NITRITATION ANAMMOX BIOFILMS

Carolina Suarez, Christopher J. Sedlacek, David J.I. Gustavsson, Alexander Eiler, Oskar Modin, Malte Hermansson, Frank Persson.

Content

SUPPLEMENTARY RESULTS AND DISCUSSION 1

SUPPLEMENTARY FIGURES 4

SUPPLEMENTARY TABLES 15

SUPPLEMENTARY REFERENCES 15

SUPPLEMENTARY RESULTS AND DISCUSSION

Nitrosomonas MAGs

Using a set of 203 single-copy genes, the taxonomic affiliation of four of these MAGs was established (Supplementary Figure 8). The MAGs SJ624 and SJ754 belong to *Nitrosomonas* cluster 7, i.e., the *Nitrosomonas europaea / mobilis* cluster. While the MAG SJ624 is most closely related to *N. europaea*, SJ754 is most similar to *N. mobilis*, although it only shares 82.2% average nucleotide identity (ANI) (Supplementary Figure 8). The MAG SJ76 is a member of *Nitrosomonas* cluster 6a (*Nitrosomonas oligotropha* cluster) sharing 99.7% ANI with *Nitrosomonas* sp. JL21, an AOB isolated from laboratory activated sludge [1]. The MAG SJ634 is a member of *Nitrosomonas* cluster 6b, a group of AOB commonly associated with estuarine and marine environments [2]. Due to its low completeness the MAG SJ328 was not included in this analysis. However, phylogenetic analysis of the ribosomal S2 gene indicates that SJ328 is closely related to SJ624 in cluster 7 (Supplementary Figure 9). All five *Nitrosomonas* MAGs contained a mix of the essential genes required for both nitrification and for denitrification to nitrous oxide (N_2O) (File S1).

The AOB SJ754 encodes two forms of complex I (NADH:quinone oxidoreductase or NADH dehydrogenase) of the electron transport chain. It encodes a canonical type I NADH dehydrogenase (*nuoA-N*), parts of which are found in all five AOB MAGs and a sodium dependent NADH:quinone oxidoreductase (type III NADH dehydrogenase), which can regulate the cytoplasmic sodium concentration. If operated in reverse, this complex would allow for the generation of NADH with

sodium motive force [3], providing an energetic advantage over AOB competitors that depend solely on proton motive force to generate cellular reducing power.

Previously characterized *N. mobilis* sp. have been observed to be halotolerant and, in some cases, even obligately halophilic [4,5]. Indeed, SJ754 contains several gene clusters that would facilitate an adaptation to saline environments (Supporting File S1). In addition, SJ754 is also the only AOB MAG to encode parts of both low and high affinity forms of RuBisCO, which may provide an advantage during times low and fluctuating CO2 concentrations. The concentration of inorganic carbon in the mainstream was more than tenfold lower than in the sidestream [6], which may explain why SJ754 had a competitive advantage in the mainstream environment of fluctuating CO_2 concentrations.

Interestingly, the AOB abundant at high sidestream exposure are expected to have very different optimal growth conditions based on their phylogeny [2,7]. Being a member of the *N. oligotropha* cluster 6a, SJ76 represents an AOB most likely adapted to low ammonium concentrations, in contrast to the other four *Nitrosomonas* MAGs. Hence, alternative metabolisms may be an important factor to explain the continuous low relative abundance of SJ76. Indeed, SJ76 and SJ634 both contain genes necessary for alternative metabolisms such as urea and hydrogen utilization. The closely related SJ624 and SJ328, belonging to *Nitrosomonas* cluster 7, both encode a possible high affinity terminal oxidase (sNOR), shown to be upregulated in AOB grown under oxygen-limiting conditions [8]. Lastly, SJ634, which belongs to *Nitrosomonas* cluster 6b, encodes a variety of genes related to halotolerance, including: 1) a sodium motive force driven type III NAD dehydrogenase, 2) a sodium motive force driven N-type ATPase [9], and 3) biosynthesis genes for the compatible solutes ectoine and hydroxyectoine [10] (File S1).

Although none of the *Nitrosomonas* MAGs included an *amoA* gene, multiple *amoA* sequences were found in the metagenomic assembly, corresponding to *Nitrosomonas* cluster 7, 6a, 6b and 8 (Supplementary Figure 10). The absence of *amoA* in the MAGs, might be due to the presence of multiple copies of *amoA* being present in the genome, as seen for *N. europaea*, [11], which makes difficult binning.

***Nitrospira* MAGs**

Four *Nitrospira* MAGs were resolved after binning (Figure 5A). Three of them are members of *Nitrospira* lineage I (SJ521, SJ669, and SJ846). The fourth is a member of *Nitrospira* lineage II (SJ32), a group that comprises both nitrite oxidizers and comammox bacteria (Supplementary Figure 11) [12,13]. Although comammox *Nitrospira* have been observed in WWTPs [14,15], a search for *amoA* in the entire metagenome assembly did not identify any *Nitrospira amoA* (Supplementary Figure 10), suggesting that comammox *Nitrospira* were not present or were below detection.

All four *Nitrospira* MAGs contained the expected genes essential for nitrite oxidation, and genes indicating a reverse TCA cycle method of carbon fixation (File S1) [16]. Although MAG SJ32 encodes many of the genes necessary for a possible functional hydrogenase, the relative abundance of SJ32 in the *Nitrospira* population was low in all treatments and decreases with increased sidestream exposure (Figure 5). Therefore, it seems that this does not provide a competitive advantage in these environments. In addition, SJ669 does not encode for all genes encoding any of these alternative metabolisms, but this is most likely due to the incompleteness of the MAG (~58%) (File S1).

Anammox MAGs

SJ692 had a 98.9% ANI to *Brocadia sapporoensis*, which had previously been identified as the dominant anammox bacterium at the Sjölunda PNA bioreactors [6], as well as in other wastewater bioreactors [17,18]. Another *Brocadia* MAG with the same abundance across samples was SJ698, which appears to be a new species of *Brocadia* (Supplementary Figure 12), although its 16S gene had a 98% identity to a sequence from the Yangtze estuary in China [19].

B. sapporoensis is a comparably fast growing anammox bacterium [20]. It has been proposed that the small genome size of *B. sapporoensis* is linked to its fast growth [21]. A genome size of 2.9 Mb was reported for *B. sapporoensis* [21], while a 2.8 Mb genome was observed for SJ692, with a 95% completion as estimated with CheckM (File S1). This was in fact smaller than two other two anammox MAGs in the PNA biofilm which both had a genome size of 3.2 MB and were estimated to be >95% complete with CheckM (File S1).

One mechanism that could allow SJ698 to compete with the dominant SJ692 is the presence of a Type VI secretion system (T6SS) in the SJ692 genome. T6SSs are used by bacteria to export proteins to neighbouring cells [22]. Thus, the T6SSs in the anammox SJ698 could be used against other bacteria [23] or might provide protection against phagocytic amoebae [24]. Selective protozoan predation of anammox bacteria has been reported before [25], and hence it is possible that anammox bacteria differ in their defence strategies against predation. In addition, we observed a cyanase in the SJ698 genome. Cyanases are uncommon among anammox bacteria, with the exception of some marine anammox bacteria in the genus *Scalindua* [26,27]. This could provide an alternative source of ammonium to SJ698.

The highest ANI for SJ251 was 99.2% to the *B. fulgida* genome. All the anammox MAGs had an acetyl-CoA synthetase, which would allow converting acetate into acetyl-CoA. However only SJ251 encoded genes for acetate kinase (*ackA*) and phosphate acetyltransferase (PTA), where acetyl-CoA is made via acetyl phosphate. In *Escherichia coli*, *ackA* + PTA are used at high acetate concentrations [28], and this agrees with a previous report of *B. fulgida* being enriched when acetate was added to the medium [29].

SUPPLEMENTARY FIGURES

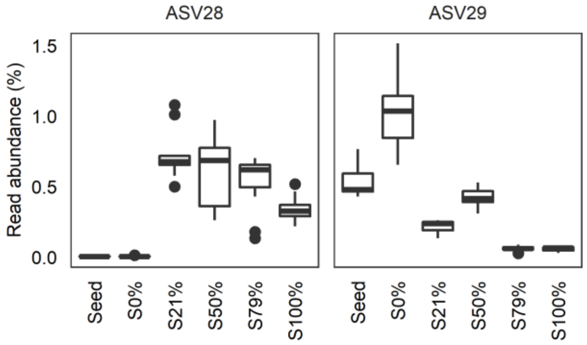

Supplementary Figure 1: Read abundances of the two most abundant *Nitrosomonas* 16S rRNA ASVs.

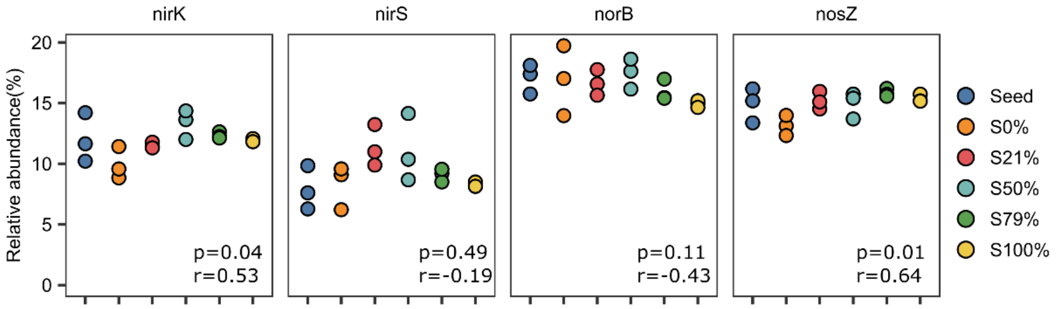

Supplementary Figure 2: Total relative abundance of MAGs with *nirK*, *nirS*, *norB* and *nosZ* genes. Values in each plot show Pearson correlation between sidestream exposure and relative abundance among samples taken in day 58.

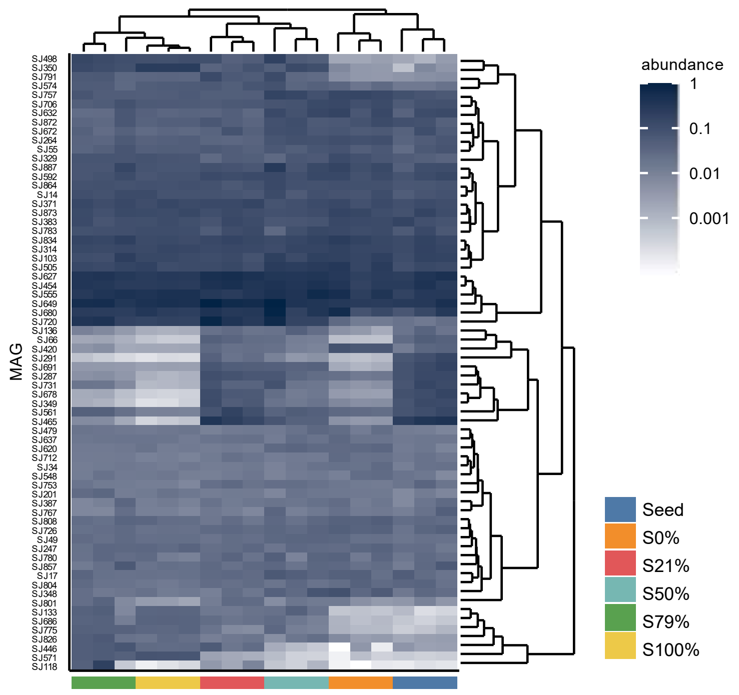

Supplementary Figure 3: Relative abundance of MAGs with the *nirS* genes. Samples and MAGs were clustered by the Bray-Curtis distance of the square transformed data.

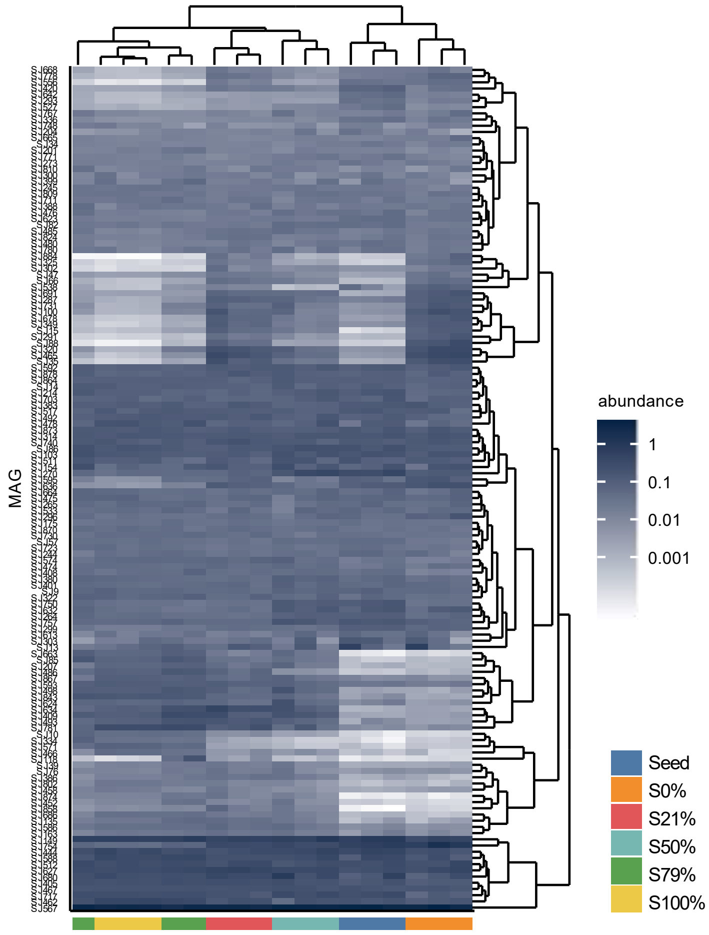

Supplementary Figure 4: Relative abundance of MAGs with a *norB* gene. Samples and MAGs were clustered by the Bray-Curtis distance of the square transformed data.

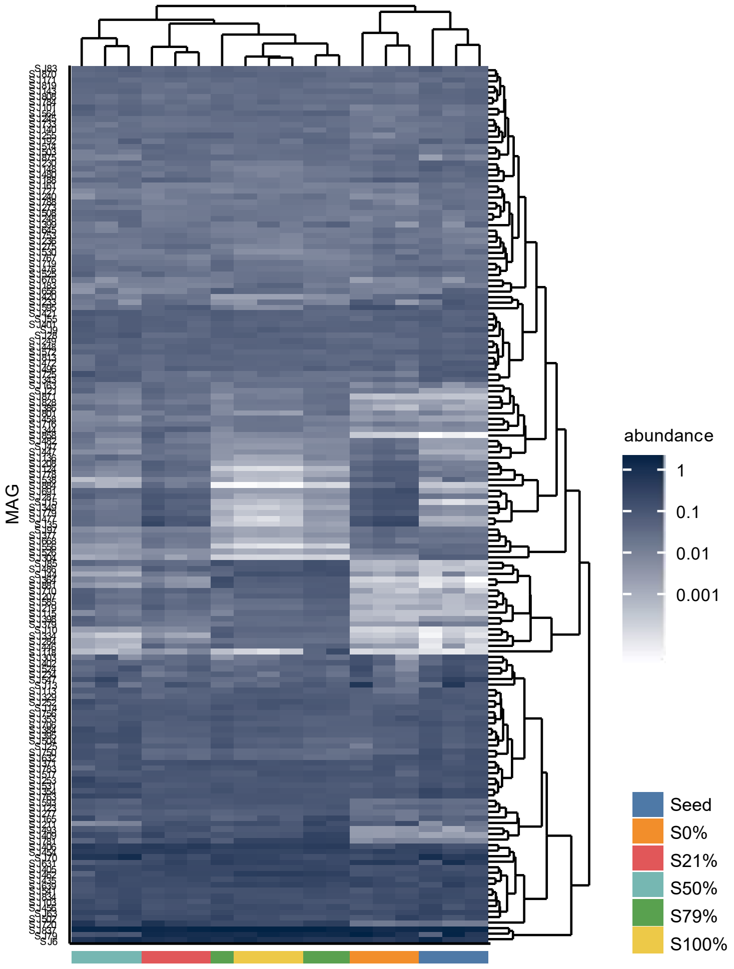

Supplementary Figure 5: Relative abundance of MAGs with a *nosZ* gene. Samples and MAGs were clustered by the Bray-Curtis distance of the square transformed data.

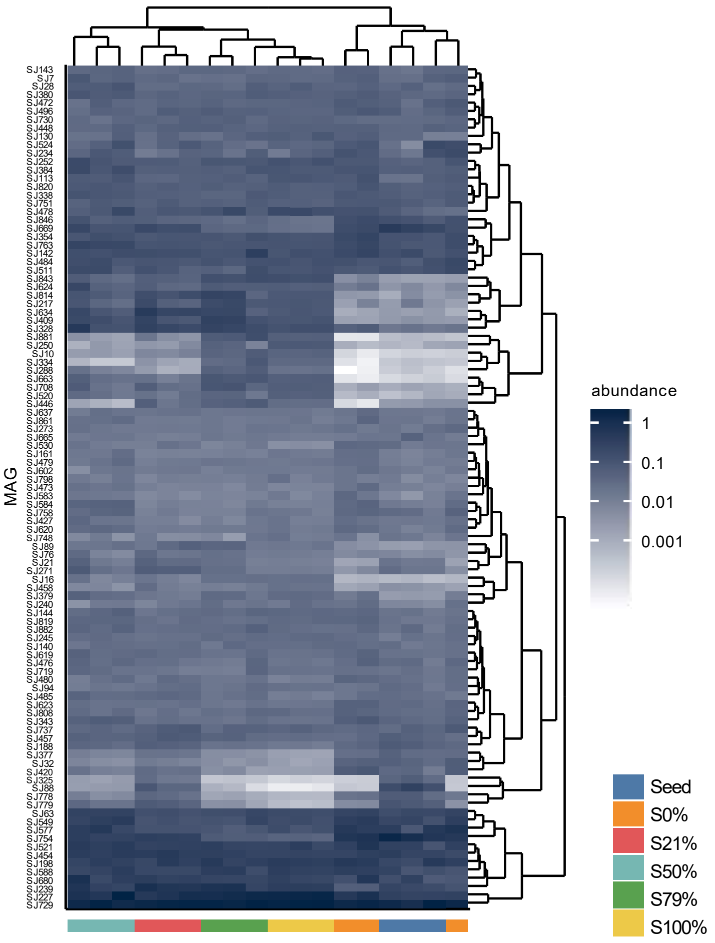

Supplementary Figure 6: Relative abundance of MAGs with a *nirK* gene. Samples and MAGs were clustered by the Bray-Curtis distance of the square transformed data.

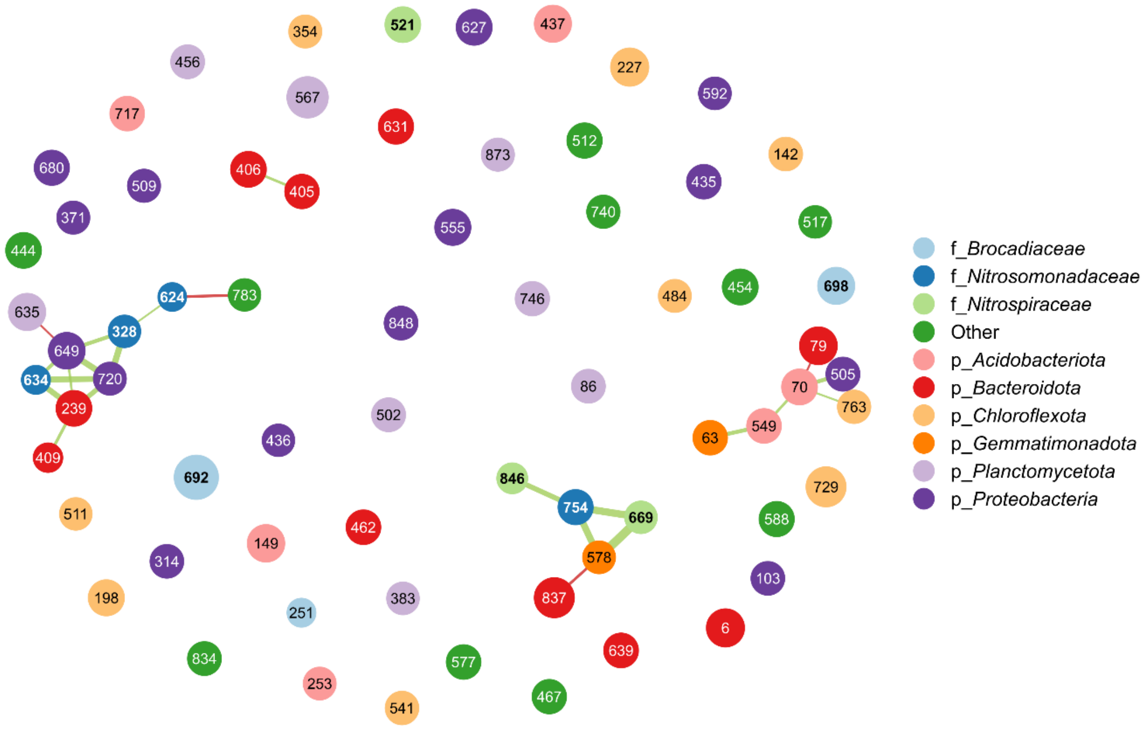

Supplementary Figure 7: Network analysis of abundant MAGs. Each circle (node) represents a MAG; connections (edges) indicate that MAG abundances are not independent of each other. Green edges show a positive link, while red edges indicate a negative link. The size of the node is proportional to its mean centred-long-ratio abundance. The thickness of an edge indicates edge weight.

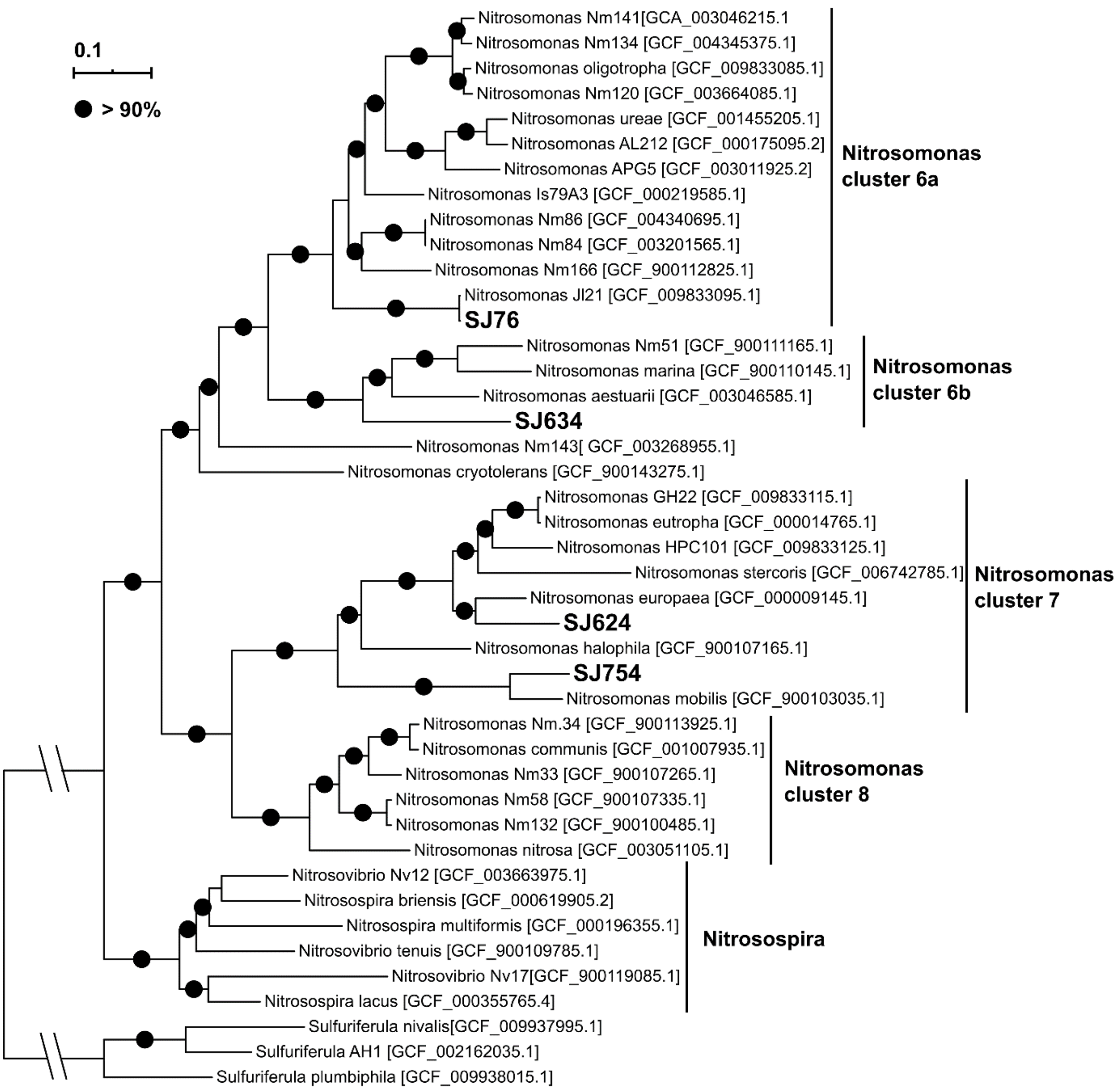

Supplementary Figure 8: Phylogenetic tree for MAGs classified as *Nitrosomonas* based on 203 single-copy genes. Circles show > 90% ultrafast bootstrap support for a clade. *Sulfuriferula* was used as outgroup.

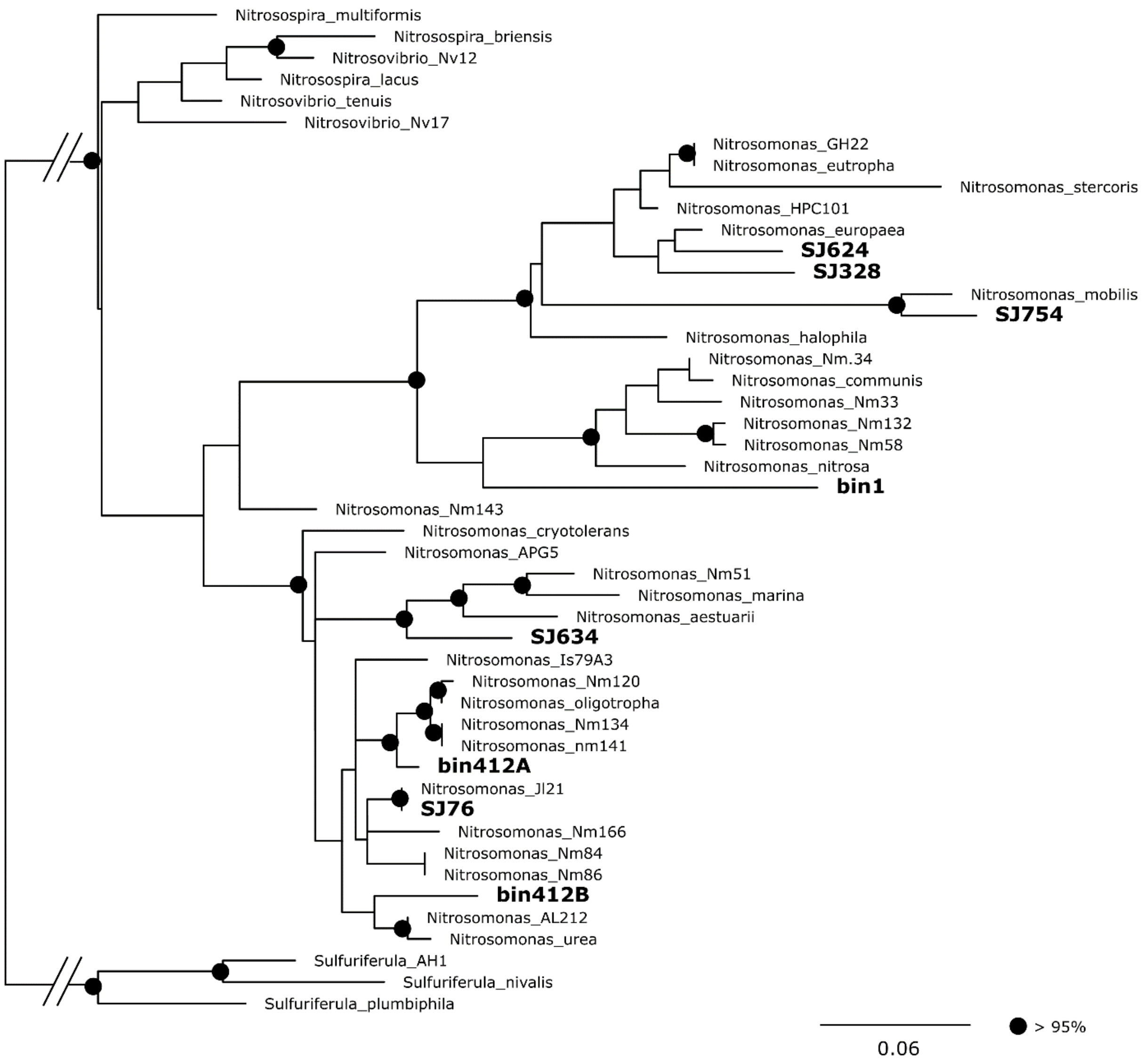

Supplementary Figure 9: Phylogenetic tree of ribosomal protein S2 in MAGs classified as *Nitrosomonas*, using a LG+R3 substitution model. Circles show > 95% ultrafast bootstrap support for a clade. *Sulfuriferula* was used as outgroup. Bin412 is a bin with high contamination, and bin1 is a bin with low completion.

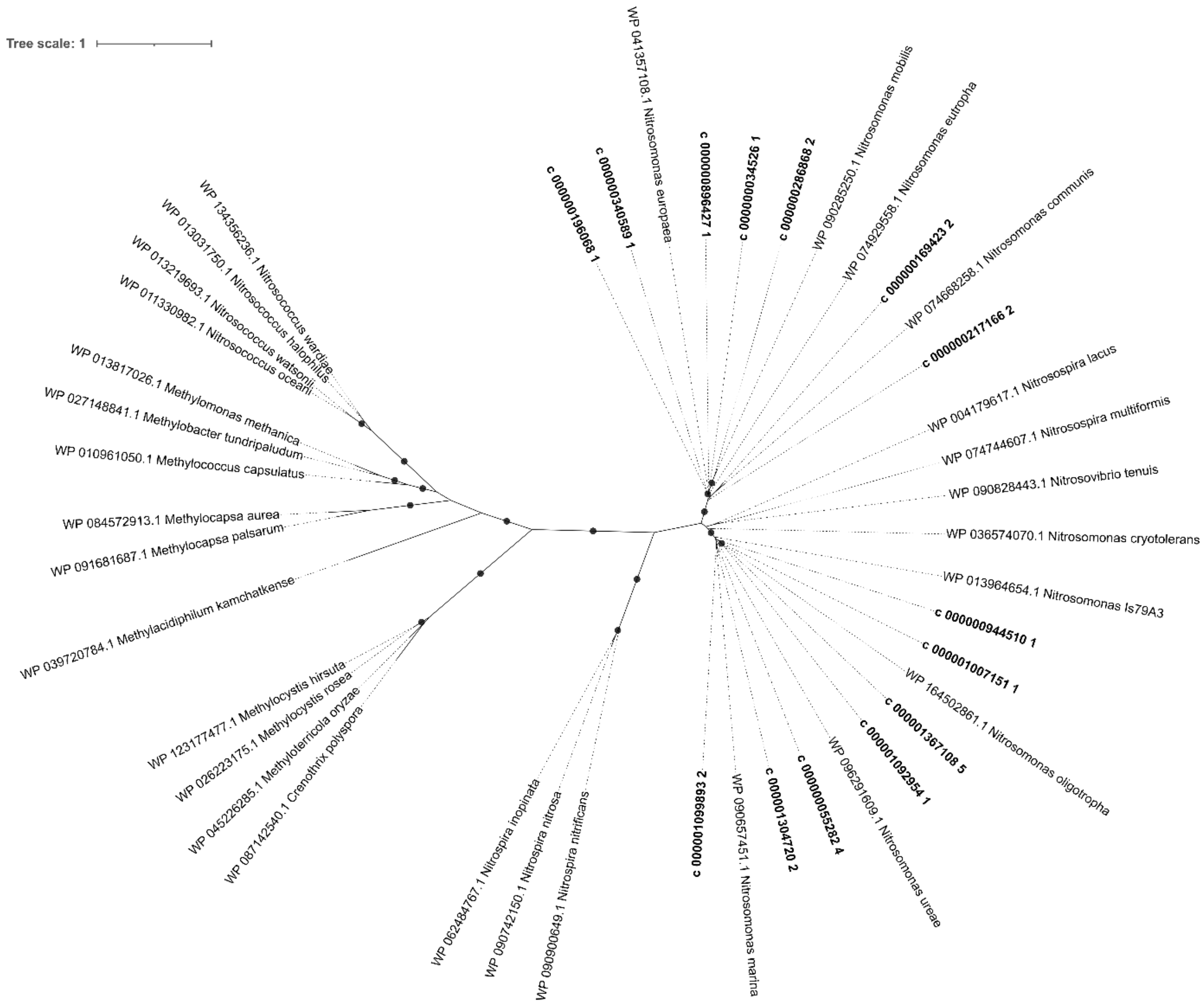

Supplementary Figure 10: Unrooted phylogenetic tree of the bacterial *amoA* and *pmoA* gene. Labels in bold indicate *amoA* sequences from this study. Circles show > 95% ultrafast bootstrap support for a clade.

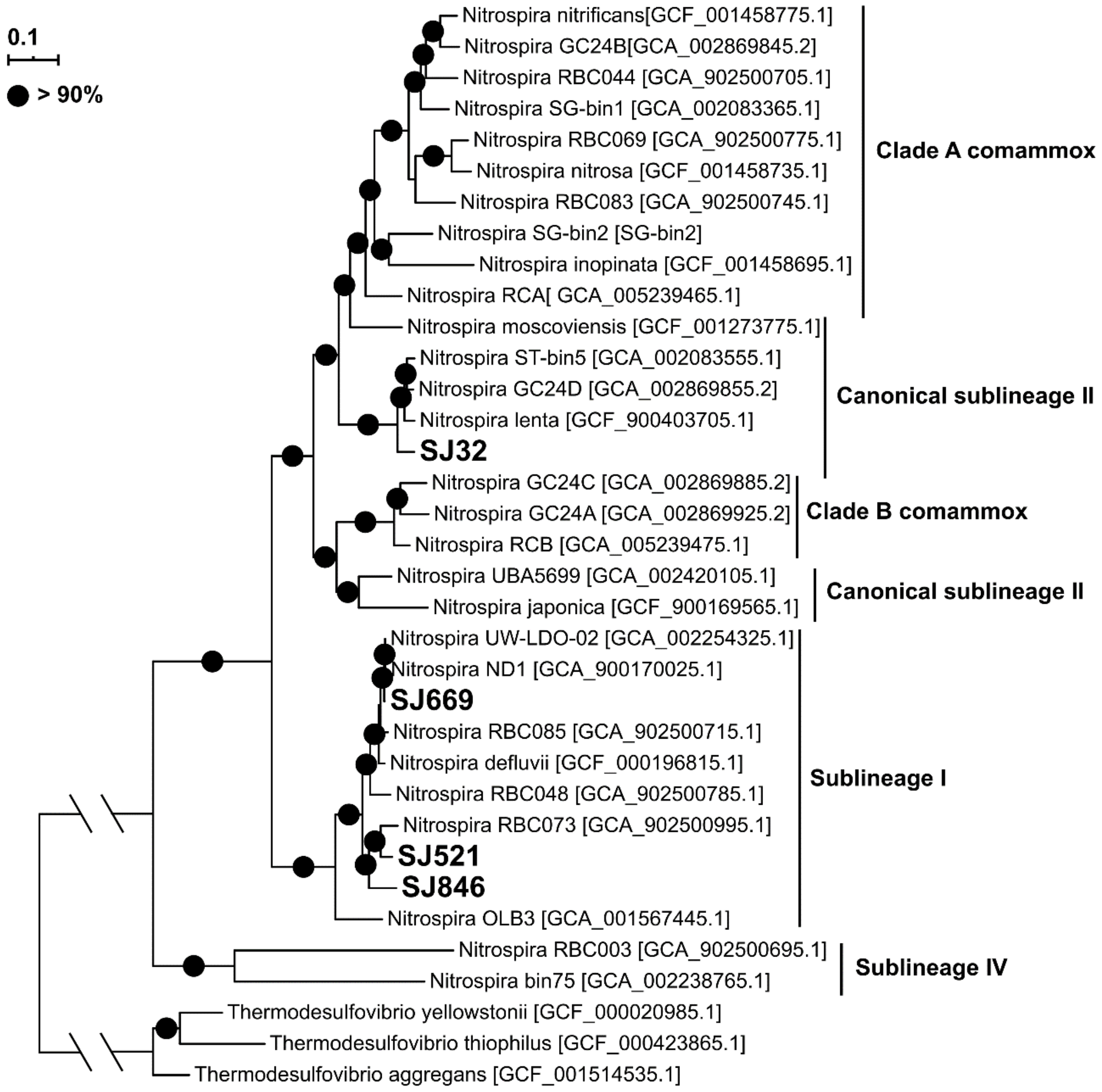

Figure 11: Phylogenetic tree for MAGs classified as *Nitrospira* based on 79 single-copy genes. Comammox clades were identified based on literature. *Thermodesulfovibrio* was used as outgroup. Circles show > 90% ultrafast bootstrap support for a clade.

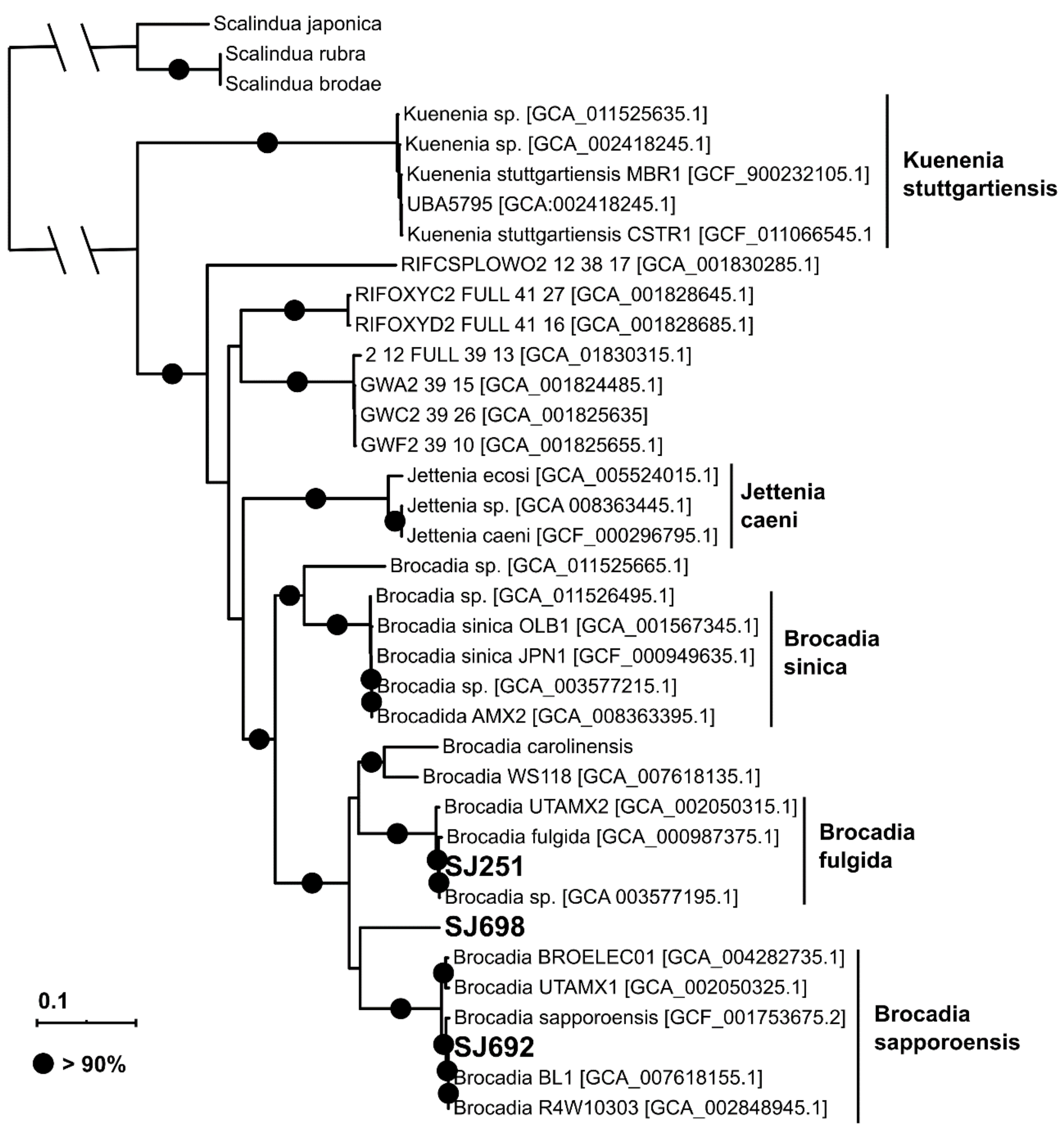

Supplementary Figure 12: Phylogenetic tree for MAGs classified as *Brocadiaceae* based on 79 single-copy genes. The marine anammox *Scalinduaceae* were used as outgroup. Circles show > 90% ultrafast bootstrap support for a clade.

SUPPLEMENTARY TABLES

Supplementary Table 1: Conductivity and concentration of ions in sidestream and mainstream wastewater at the Sjölunda WWTP.

**Sample**	**Date**	**Conductivity**	**Na^+**	**Cl^-**	**NH_4^+**	**K^+**	**PO_4^{3-}**	**Mg^{2+}**	**Ca^{2+}**	**SO_4^{2-}**
	YYMMDD	mS/cm	mM	mM	mM	mM	mM	mM	mM	mM
Mainstream	210117	1.23	3.99	2.92	1.06	0.39	0.63	0.79	1.86	1.05
Mainstream	210118	1.23	6.21	4.06	1.69	0.52	0.86	1.00	2.34	1.20
Mainstream	210119	1.38	7.67	4.89	1.96	0.56	0.76	0.91	2.48	1.01
Sidestream	210118	6.97	5.74	3.79	64.6	2.69	1.43	1.98	3.70	0.50
Sidestream	210119	7.32	5.62	3.80	70.5	2.77	1.39	1.94	4.14	0.12
Sidestream	210120	7.13	6.58	4.37	68.6	2.93	1.51	2.06	3.99	0.28

SUPPLEMENTARY REFERENCES

1 Suwa, Y., Sumino, T. & Noto, K. Phylogenetic relationships of activated sludge isolates of ammonia oxidizers with different sensitivities to ammonium sulfate. *The Journal of General and Applied Microbiology* **43**, 373-379 (1997).

2 Prosser, J. I., Head, I. M. & Stein, L. Y. in *The Prokaryotes: Alphaproteobacteria and Betaproteobacteria* (eds Eugene Rosenberg *et al.*) 901-918 (Springer Berlin Heidelberg, 2014).

3 Verkhovsky, M. I. & Bogachev, A. V. Sodium-translocating NADH:quinone oxidoreductase as a redox-driven ion pump. *Biochimica et Biophysica Acta (BBA) - Bioenergetics* **1797**, 738-746 (2010).

4 Koops, H.-P., Harms, H. & Wehrmann, H. Isolation of a moderate halophilic ammonia-oxidizing bacterium, Nitrosococcus mobilis nov. sp. *Archives of Microbiology* **107**, 277-282 (1976).

5 Thandar, S. M., Ushiki, N., Fujitani, H., Sekiguchi, Y. & Tsuneda, S. Ecophysiology and comparative genomics of Nitrosomonas mobilis Ms1 isolated from autotrophic nitrifying granules of wastewater treatment bioreactor. *Frontiers in Microbiology* **7**, 1869 (2016).

6 Gustavsson, D. J. I., Suarez, C., Wilén, B.-M., Hermansson, M. & Persson, F. Long-term stability of partial nitritation-anammox for treatment of municipal wastewater in a moving bed biofilm reactor pilot system. *Science of the Total Environment*, 136342 (2020).

7 Koops, H.-P. & Pommerening-Röser, A. in *Bergey's Manual® of Systematic Bacteriology: Volume Two: The Proteobacteria, Part A Introductory Essays* (eds Don J. Brenner, Noel R. Krieg, James T. Staley, & George M. Garrity) 141-147 (Springer US, 2005).

8 Sedlacek, C. J. *et al.* Transcriptomic response of Nitrosomonas europaea transitioned from ammonia- to oxygen-limited steady-state growth. *mSystems* **5**, e00562-00519 (2020).

9 Soontharapirakkul, K. *et al.* Halotolerant cyanobacterium Aphanothece halophytica contains an Na^{+}-dependent F1F0-ATP synthase with a potential role in salt-stress tolerance. *Journal of Biological Chemistry* **286**, 10169-10176 (2011).

10 Czech, L. *et al.* Role of the extremolytes ectoine and hydroxyectoine as stress protectants and nutrients: genetics, phylogenomics, biochemistry, and structural analysis. *Genes* **9,** 177 (2018).

11 Hommes, N. G., Sayavedra-Soto, L. A. & Arp, D. J. Transcript analysis of multiple copies amo (encoding Ammonia Monooxygenase) and hao (encoding Hydroxylamine Oxidoreductase) in Nitrosomonas europaea. *Journal of Bacteriology* **183**, 1096 (2001).

12 van Kessel, M. A. H. J. *et al.* Complete nitrification by a single microorganism. *Nature* **528**, 555-559 (2015).

13 Daims, H. *et al.* Complete nitrification by Nitrospira bacteria. *Nature* **528**, 504–509 (2015).

14 Roots, P. *et al.* Comammox Nitrospira are the dominant ammonia oxidizers in a mainstream low dissolved oxygen nitrification reactor. *Water Research* **157**, 396-405 (2019).

15 Spasov, E. *et al.* High functional diversity among Nitrospira populations that dominate rotating biological contactor microbial communities in a municipal wastewater treatment plant. *The ISME Journal* (2020).

16 Lücker, S. *et al.* A Nitrospira metagenome illuminates the physiology and evolution of globally important nitrite-oxidizing bacteria. *Proceedings of the National Academy of Sciences U.S.A.* **107**, 13479 (2010).

17 Persson, F. *et al.* Community structure of partial nitritation-anammox biofilms at decreasing substrate concentrations and low temperature. *Microbial Biotechnology* **10**, 761-772 (2017).

18 Park, H. *et al.* Impact of inocula and growth mode on the molecular microbial ecology of anaerobic ammonia oxidation (anammox) bioreactor communities. *Water Research* **44**, 5005-5013 (2010).

19 Hou, L. *et al.* Anaerobic ammonium oxidation (anammox) bacterial diversity, abundance, and activity in marsh sediments of the Yangtze Estuary. *Journal of Geophysical Research: Biogeosciences* **118**, 1237-1246 (2013).

20 Lotti, T., Kleerebezem, R., Abelleira-Pereira, J. M., Abbas, B. & van Loosdrecht, M. C. M. Faster through training: The anammox case. *Water Research* **81**, 261-268 (2015).

21 Ali, M. *et al.* Draft genome sequence of the anaerobic ammonium-oxidizing bacterium "Candidatus Brocadia sp. 40". *Genome Announcements* **4**, e01377-01316 (2016).

22 Coulthurst, S. J. The Type VI secretion system – a widespread and versatile cell targeting system. *Research in Microbiology* **164**, 640-654 (2013).

23 Hood, R. D. *et al.* A Type VI secretion system of Pseudomonas aeruginosa targets a toxin to Bacteria. *Cell Host & Microbe* **7**, 25-37 (2010).

24 Zheng, J., Ho, B. & Mekalanos, J. J. Genetic analysis of anti-amoebae and anti-bacterial activities of the type VI secretion system in Vibrio cholerae. *PLOS ONE* **6**, e23876 (2011).

25 Suarez, C., Persson, F. & Hermansson, M. Predation of nitritation–anammox biofilms used for nitrogen removal from wastewater. *FEMS Microbiology Ecology* **91**, fiv124 (2015).

26 Ganesh, S. *et al.* Single cell genomic and transcriptomic evidence for the use of alternative nitrogen substrates by anammox bacteria. *The ISME Journal* **12**, 2706-2722 (2018).

27 Zhao, R. *et al.* Geochemical transition zone powering microbial growth in subsurface sediments. *Proceedings of the National Academy of Sciences U.S.A.* **117**, 32617-32626 (2020).

28 Kumari, S., Tishel, R., Eisenbach, M. & Wolfe, A. J. Cloning, characterization, and functional expression of acs, the gene which encodes acetyl coenzyme A synthetase in Escherichia coli. *Journal of Bacteriology* **177**, 2878 (1995).

29 Kartal, B. *et al.* Candidatus 'Brocadia fulgida': an autofluorescent anaerobic ammonium oxidizing bacterium. *FEMS Microbiology Ecology* **63**, 46-55 (2008).
