# Supplementary material for: Disturbance-based management of ecosystem services and disservices in partial nitritation-anammox biofilms
Source: NPJ Biofilms Microbiomes. 2022 Jun 8;8:47. doi: 10.1038/s41522-022-00308-w (PMC9178042; doi:10.1038/s41522-022-00308-w)
Supplement: Supplementary file 3 — Reporting Summary Checklist [file 41522_2022_308_MOESM3_ESM.pdf]

nature portfolio

Corresponding author(s): Frank Persson

Last updated by author(s): May 2, 2022

Reporting Summary

Nature Portfolio wishes to improve the reproducibility of the work that we publish. This form provides structure for consistency and transparency in reporting. For further information on Nature Portfolio policies, see our Editorial Policies and the Editorial Policy Checklist.

Statistics

For all statistical analyses, confirm that the following items are present in the figure legend, table legend, main text, or Methods section.

n/a	Confirmed	
☐	☒	The exact sample size (*n*) for each experimental group/condition, given as a discrete number and unit of measurement
☐	☒	A statement on whether measurements were taken from distinct samples or whether the same sample was measured repeatedly
☐	☒	The statistical test(s) used AND whether they are one- or two-sided<br>*Only common tests should be described solely by name; describe more complex techniques in the Methods section.*
☐	☒	A description of all covariates tested
☒	☐	A description of any assumptions or corrections, such as tests of normality and adjustment for multiple comparisons
☐	☒	A full description of the statistical parameters including central tendency (e.g. means) or other basic estimates (e.g. regression coefficient) AND variation (e.g. standard deviation) or associated estimates of uncertainty (e.g. confidence intervals)
☐	☒	For null hypothesis testing, the test statistic (e.g. *F*, *t*, *r*) with confidence intervals, effect sizes, degrees of freedom and *P* value noted<br>*Give P values as exact values whenever suitable.*
☒	☐	For Bayesian analysis, information on the choice of priors and Markov chain Monte Carlo settings
☒	☐	For hierarchical and complex designs, identification of the appropriate level for tests and full reporting of outcomes
☒	☐	Estimates of effect sizes (e.g. Cohen's *d*, Pearson's *r*), indicating how they were calculated

Our web collection on statistics for biologists contains articles on many of the points above.

Software and code

Policy information about availability of computer code

Data collection	Amplicon seq (16S): DADA2 v1.16, SILVA 138. Shotgun seq: Trimmomatic v0.39 , Megahit 1.29, Bowtie2 v2.3.5, Prodigal v.2.6.3.
Data analysis	SingleM v0.13.2, Hilldiv v1.5.1, Kaiju v1.7.3, DIAMOND v2.0.4, MCL v14-137, HMMER 3.3, MetaBAT2 v2.12.1, CheckM v1.0.12, GTDB-Tk v1.0.2, GtoTree v1.2.1, IQ-TREE v.2.0.3, ModelFinder v (this one comes with IQ-TREE), FastANI v1.3.1, eggNOG-mapper v1.0.3, Genoscope.

For manuscripts utilizing custom algorithms or software that are central to the research but not yet described in published literature, software must be made available to editors and reviewers. We strongly encourage code deposition in a community repository (e.g. GitHub). See the Nature Portfolio guidelines for submitting code & software for further information.

Data

Policy information about availability of data

All manuscripts must include a data availability statement. This statement should provide the following information, where applicable:

- Accession codes, unique identifiers, or web links for publicly available datasets
- A description of any restrictions on data availability
- For clinical datasets or third party data, please ensure that the statement adheres to our policy

Amplicon sequencing reads, raw shotgun metagenomics reads, and metagenome assembled genomes (MAGs) are available at NCBI under the bioproject PRJNA611787. All data generated or analysed during this study will be available upon request to the corresponding author.

Field-specific reporting

Please select the one below that is the best fit for your research. If you are not sure, read the appropriate sections before making your selection.

☐ Life sciences ☐ Behavioural & social sciences ☒ Ecological, evolutionary & environmental sciences

For a reference copy of the document with all sections, see nature.com/documents/nr-reporting-summary-flat.pdf

Ecological, evolutionary & environmental sciences study design

All studies must disclose on these points even when the disclosure is negative.

Study description	This is a study on how PNA biofilms from a bioreactor treating mainstream wastewater are affected by a disturbance regimen (various levels of exposure to sidestream wastewater) to alter the biofilm community. Biofilms on individual biofilm carriers from one reactor (time zero) was subjected to five different levels of disturbance (five treatments) for 58 days. Replicate biofilm carriers were analysed (nine replicates for amplicon seq, thre replicates for shotgun seq) for day zero and the five treatments (after 58 days).
Research sample	Biofilms from a partial nitritation-anammox pilot plant.
Sampling strategy	Biofilms were sampled at the study beginning (time zero) and end (58 days). DNA was extracted from individual biofilm carriers representing replicates from each sample. Previous experiences of biofilm carriers in the same system were used to determine the number of replicates. A total number of 12 biofilm carriers were individually extracted from each sample. All DNA extracts were used for amplicon sequencing (16S rRNA gene) and a subset (three per treatment) for shotgun sequencing.
Data collection	Samples were collected by CS (first author) and FP (corresponding). Subsequent wet lab work (DNA extraction, purification etc.) was carried out by CS.
Timing and spatial scale	Samples were collected before (day zero) and after the treatment (58 days). A pre-study (unpublished) had indicated the period (58 days) of being an appropriate duration to achieve clear results. The reason for the study design was to compare multiple treatments with sufficient replication.
Data exclusions	A total of 12 DNA extracts per sample were initially prepared for 16S rRNA gene amplicon sequencing. However, the quality of the sequences for three replicates within one sample was too low to be included. Hence, thee replicates from each treatment were randomly omitted to maintain a balanced design. The number of replicates left (nine) was still robust enough to maintain statistical power.
Reproducibility	A pre-study (unpublished) resulted in findings similar to the actual study, although not measured at the same level of detail.
Randomization	Sampling consisted of random picking of biofilm carriers from each treatment (a cage with biofilm carriers).
Blinding	Blinding was not used during the acquisition and analysis of data (bioinformatics and statistical analyses). During sampling, blinding was simply impossible but also unnecessary as the biofilm carriers look identical to the human eye. Since all samples are analysed in batches, blinding was seen as unnecessary, as no special attention was given to any specific samples during data analysis.

Did the study involve field work? ☐ Yes ☒ No

Reporting for specific materials, systems and methods

We require information from authors about some types of materials, experimental systems and methods used in many studies. Here, indicate whether each material, system or method listed is relevant to your study. If you are not sure if a list item applies to your research, read the appropriate section before selecting a response.

Materials & experimental systems

n/a	Involved in the study	
☒	☐	Antibodies
☒	☐	Eukaryotic cell lines
☒	☐	Palaeontology and archaeology
☒	☐	Animals and other organisms
☒	☐	Human research participants
☒	☐	Clinical data
☒	☐	Dual use research of concern

Methods

n/a	Involved in the study	
☒	☐	ChIP-seq
☒	☐	Flow cytometry
☒	☐	MRI-based neuroimaging
